# Post-exposure prevention in Marburg virus outbreaks: potential role of remdesivir in emergency response

**DOI:** 10.1097/MS9.0000000000004037

**Published:** 2025-10-09

**Authors:** Christian Tague, Joshua Ekouo, Dujardin Makeda, Hermann Yokolo, Jones Onesime, Styves Banga, Alimasi Kashafali, Aymar Akilimali

**Affiliations:** Department of Research, Medical Research Circle (MedReC), Goma, DR Congo

**Keywords:** epidemics, Marburg virus, prevention, remdesivir

## Abstract

Marburg virus (MARV), a close relative of the Ebola virus, causes severe hemorrhagic fevers with a case fatality rate of up to 88%. In the absence of specific treatment or vaccine, its rapid spread through epidemics in Africa, particularly in Angola, DRC, Guinea, Uganda, and recently Tanzania, poses a major threat to global public health. The use of post-exposure prevention, already effective against viruses such as HIV and rabies, could offer a promising strategy to interrupt infection in recently exposed individuals. Remdesivir, a nucleotide analog initially developed against the Ebola virus and used for SARS-CoV-2, has shown significant antiviral activity against MARV in animal models. Administered within 24–48 hours of exposure, it reduces viral load and improves survival. This asymptomatic but active window of viral replication provides a valuable opportunity for intervention. Although clinical results are still lacking, preclinical data support urgent consideration of remdesivir as a rapid response tool. However, its logistical constraints (injectable form) limit its deployment in remote areas. Nevertheless, its integration into emergency protocols, accompanied by investments in research, early authorizations, and strategic stockpiles, could strengthen response capacities. While awaiting a vaccine, each antiviral option deserves rigorous attention to limit transmission and mortality during future Marburg outbreaks.

Marburg virus (MARV), in the family *Filoviridae*, is a highly lethal pathogen responsible for severe hemorrhagic fevers in humans. A close cousin of the Ebola virus, it was first identified in 1967 during simultaneous outbreaks in Marburg and Frankfurt, Germany, and in Belgrade, Serbia, following exposure to infected primates imported from Uganda^[1]^. Since then, several outbreaks have been documented in Africa, including Angola, Uganda, Guinea, the Democratic Republic of Congo, and, more recently, Tanzania, highlighting its epidemic potential in regions with limited health infrastructure. With a case fatality rate ranging from 24% to 88% depending on the epidemic, and human-to-human transmission facilitated by contact with bodily fluids, MARV poses a major threat to global public health. Currently, no specifically approved antiviral treatment or vaccine is available for MARV. In this context, post-exposure prevention (PEP), already successfully used for other serious viral infections (rabies, HIV, and hepatitis B), appears as a potential strategy to interrupt the progression of infection in recently exposed individuals^[1,2]^. Remdesivir, a nucleotide analog initially developed against RNA viruses such as Ebola and SARS-CoV-2, has demonstrated significant in antiviral activity both in vitro and in vivo in animal models against MARV. Its use in post-exposure prevention is currently of increasing interest, particularly in rapid outbreak response scenarios against a broad spectrum of RNA viruses, including MARV, Ebola, SARS-CoV, MERS-CoV, Nipah, and Lassa virus, whereas many monoclonal antibodies (mAbs) are virus-specific and thus more limited in scope^[2,3]^. Recent studies have identified several promising candidates for PEP against MARV, although most data are derived from nonhuman primate (NHP) models. Two notable investigational options include obeldesivir (ODV) and mAbs. ODV, an oral nucleoside analog prodrug, offers logistical advantages due to its oral formulation. In a 2025 study, 80% of cynomolgus macaques (*n* = 6) survived a 1000-fold lethal MARV challenge when treatment began 24 hours post-exposure and continued daily for 10 days^[4,5]^. On the other hand, mAbs, when administered alone, showed modest efficacy. However, combination therapy with mAbs and remdesivir significantly improved outcomes. In rhesus macaques, initiating this combination later in the disease course resulted in 80% survival, indicating a synergistic effect between viral neutralization by mAbs and viral replication inhibition by remdesivir^[5–7]^. Remdesivir alone also showed a clear survival advantage. In a pivotal study, 83% of macaques survived when treated with a 10 mg/kg loading dose followed by 5 mg/kg daily, starting on day 5 post-exposure, compared to 0% survival in untreated controls. These results suggest that remdesivir maintains high efficacy even when administered at later stages of infection, unlike many other PEP options. It is important to acknowledge that these results are from animal studies, and caution is needed when extrapolating to humans due to differences in physiology, disease progression, and immune responses^[3–5,7]^.

This editorial advocates for scientific and regulatory mobilization to evaluate and, potentially, integrate this approach into strategies for managing filovirus epidemics. This article complies with the TITAN 2025 guidelines – repressing the reporting and use of AI^[8]^.

MARV is naturally harbored by certain fruit bats (*Rousettus aegyptiacus*) and can be transmitted to humans through direct contact with fluids from infected animals. After human contamination, it spreads through close contact with biological fluids, particularly in healthcare settings and during funeral rites, exposing caregivers and relatives^[9]^. The incubation period, typically 2–21 days, provides a critical window for considering PEP. This phase precedes the onset of symptoms but may already coincide with active viral replication, making early intervention critical. Animal models have demonstrated that remdesivir, administered within 24–48 hours of exposure, can significantly reduce viral load and improve survival^[2,9]^. In the context of an epidemic, where every hour counts, this asymptomatic period represents a precious opportunity to act quickly and limit the cascade of transmission.

Originally developed to combat the Ebola virus, remdesivir works by inhibiting viral RNA polymerase, thereby blocking the replication of RNA viruses. Its efficacy has been confirmed against various pathogens, including SARS-CoV-2, but also MARV in several preclinical studies^[1,9,10]^. In primates exposed to MARV, administration of remdesivir within 24–48 hours post-exposure prevented the onset of symptoms and significantly increased survival^[10,11]^. A 2020 preclinical study showed that 100% of untreated macaques infected with MARV died. In contrast, 83% of animals treated with a remdesivir loading dose (10 mg/kg) survived, while 50% of those receiving 5 mg/kg survived, showing clinical improvement and reduced viral load^[4]^. These results, although limited to animal models, suggest a potentially decisive effect in a PEP strategy. Remdesivir faces major obstacles such as its injectable form, high cost, cold chain requirements, long treatment duration, lack of human data, risk of resistance, and low community acceptance^[9,11]^.

In conclusion, given the recurring threats of MARV and the lack of approved treatment, remdesivir represents a credible avenue for targeted PEP. Although clinical evidence remains to be consolidated, experimental data fully justify urgent exploration of its integration into rapid response protocols. Investing in studies, early approvals, and strategic stockpiling of remdesivir could provide a valuable cushion during future outbreaks. While we wait for effective vaccines, every available antiviral tool deserves serious evaluation and advance planning.

## Data Availability

Not applicable.
